# A Cancer Cell Cluster Marked by LincRNA *MEG3* Leads Pancreatic Ductal Adenocarcinoma Metastasis

**DOI:** 10.3389/fonc.2021.656564

**Published:** 2021-05-13

**Authors:** Hong Pan, Huanrong Diao, Wen Zhong, Taifang Wang, Ping Wen, Chunli Wu

**Affiliations:** Department of Radiation Oncology, The Fourth Affiliated Hospital of China Medical University, Shenyang, China

**Keywords:** pancreatic ductal adenocarcinoma, single cell RNA-seq, LincRNA, *MEG3*, epithelial–mesenchymal transition

## Abstract

Pancreatic ductal adenocarcinoma (PDAC) is a highly devastating disease with poor prognosis and rising incidence worldwide. Late detection and particularly aggressive characteristics are the major challenges that lead to therapeutic failure of this disease. A well described gene program and core regulators are yet to be discovered to drive the metastasis of the PDAC cells. As the development of single cell omics technologies including single cell RNA-sequencing (scRNA-seq), detailed characterization of the cellular composition of solid tumors and their microenvironments are well elaborated. In the current study, we accessed a recently published scRNA-seq dataset on primary and metastatic PDAC tissues and subset the tumor cells. By comparative analysis, we profiled the differentially expressed gene programs of primary and metastatic PDAC and found several long intergenic non-coding RNAs (LincRNAs) in top genes. The PDAC cancer cells showed some heterogeneity and were divided into four major subclusters based on gene profiles, one of which was mostly contributed by metastatic PDAC. Interestingly, this subcluster was remarkably marked by one of the above LincRNAs, *MEG3*, and exhibited significantly increased Epithelial–Mesenchymal-Transition (EMT) signatures. Ingenuity Pathway Analysis (IPA) on the signature genes of this subcluster gave multiple cancer metastasis associated and EMT signaling pathways, suggesting a critical role of this cluster in leading tumor cell metastasis. Taken together, this study displayed a PDAC cancer subcluster and its marker gene, biologically targeting of which might significantly attenuate the metastasis of tumor and might be a potential strategy for the therapeutic treatment of cancer.

## Introduction

Pancreatic ductal adenocarcinoma (PDAC) is the most prevalent neoplastic disease of the pancreas and accounts for more than 90% of all pancreatic malignancies (1). To now, PDAC has a 5-year overall survival of less than 8% and is the fourth most frequent cause of cancer-related deaths worldwide (2). The incidence of PDAC is expected to rise further in the future, and projections indicate a more than two-fold increase in the number of cases within the next ten years according to new diagnoses and PDAC-related deaths in American and European countries (3, 4). The outcome of PDAC therapeutic treatment largely relies on the stage of disease at the time of diagnosis. Traditional treatment like surgical resection followed by adjuvant chemotherapy is the only possibly curative therapy available up to now, but only 10–20% of PDAC patients present with resectable PDAC stages, and 80–90% show locally advanced, non-resectable stages or distant metastases (5, 6). Due to the increasing incidence, high mortality and less effective treatment of this disease, better research and therapeutic technologies and targets are highly needed.

PDAC is a complex, heterogeneous, and genetically unstable disease (7) with undetermined developmental and metastatic mechanisms. PDAC is thought to arise from acinaductal metaplasia and develop into invasive carcinoma through pancreatic intraepithelial neoplasia lesions as key genetic mutations accumulate (8–10). However, due to the complex components of tumor and tumor environment, it is challenging to detailly identify these ectopic genetic accumulations in cancer (11–13). Thanks to the application of single cell omics technologies in cancer study in recent years, increasing studies are profiling and landscaping the components of solid organ diseases including tumors and validating canonical gene markers and revealing novel targets and mutations (14, 15). By using of the single cell RNA-seq, PDAC tumor mass is found to be highly heterogeneous and is composed of diverse malignant and stromal cell types, which is true in both human diseases and mouse models (16, 17). Also, single-cell transcriptome analysis of tumor-associated stromal, immune, endothelial and fibroblast cells identifies signals that may support tumor development, as well as the recruitment and education of immune cells, which is consistent with the early, premalignant formation of an immunosuppressive environment mediated by interactions between acinar metaplastic cells and other cell types in the microenvironment (18).

These above studies are fully informative and deepen our understanding of the genetic alterations of tumor metaplastic cell from normal epithelial cells, however, most of the studies are based on the primary tumor and corresponding normal tissues and is not contributing the fields in defining tumor malignant grades. A most recent study profiled and compared the primary and metastatic PDAC and landscaped the cell components of both tumor grades (19). By accessing their dataset, we subset and integrate the tumor cells from primary and metastatic PDAC tumor cells. Comparative analysis illustrates the differentially expressed gene programs between primary and metastatic PDAC tumor cells. More importantly, the tumor cells are divided into four major subclusters and most are identifiable by specific LincRNAs. One of the clusters show high proliferation potentials. Another cluster is mostly derived from metastatic tumors and is specifically marked by LincRNA, *MEG3*. Gene profiling and IPA analysis suggest that this cluster shows decreased epithelial features and increased mesenchymal features, suggesting a EMT potential in this cluster. Our study determines the heterogeneity of the primary and PDAC tumor cells and reveals that *MEG3* positive tumor cells are leading the metastasis of PDAC. This cancer cell cluster might be of high malignance and targeting of them might be a potential novel therapeutic strategy for the treatment of PDAC.

## Materials and Methods

### scRNA-seq Data Accessibility

PDAC tumor specimen dissociation and single-cell RNA sequencing were previously described by data authors (19). We accessed the processed data of scRNA-seq from GEO database (Accession # GSE154778) and raw data of the scRNA-seq from SRA database (Accession # SRP272677) for our analysis. scRNA-seq data of genetically engineered mouse models of PDAC were accessed from GEO database (GSE125588) and raw data from SRA data base (PRJNA516878) (17).

### scRNA-seq Sample Integration and Quality Control (QC)

10× Matrix data for 10 primary PDAC samples and 6 metastatic samples was read by R software package Seurat (20) and Seurat object was generated based on the Matrix data. Each sample object was grouped by either sample individuals or their disease status (“Primary” or “Metastasis”). After each sample object was converted to log scale using the ‘NormalizeData’ function and highly variable genes were selected with the ‘FindVariableGenes’ function, we then identified anchors using the “FindIntegrationAnchors” function, which took a list of all the Seurat objects as input and used these anchors to integrate all the datasets together with “IntegrateData”. In the integrated Seurat object, total number of genes detected each cell, nFeature_RNA, number of transcripts each cell, nCount_RNA, and percentage of mitochondrial genes each cell, percent.MT, of each sample were visualized by violin plots. Doublets were identified as outliers with a dramatically higher number of detected genes or transcript per cell than the median. Quality control (QC) was performed by removing low-quality cells and doublets by the cell subset at: nCount_RNA <100,000 & nFeature_RNA <7,500 & percent.mt <40.

### scRNA-seq Cell Clustering

After QC, these data were converted to z-scores using the ‘ScaleData’ command. Next, we calculated principal components the “RunPCA” on the scaled data and performed linear dimensional reduction on the object. To cluster the cells, we next applied modularity optimization techniques to iteratively group cells together. The “FindNeighbors” and “FindClusters” functions implemented this procedure and contained a resolution parameter that sets the ‘granularity’ of the downstream clustering, with increased values leading to a greater number of clusters. Then we run non-linear dimensional reduction by “RunUMAP” function to visualize and explored the dataset. Cells within the graph-based clusters should co-localize on these dimension-reduction plots.

### scRNA-seq Cell Type Definition and Differentially Expressed Gene Identification

Canonical cell type marker gene expression was visualized by UMAP: EPCAM, KRT19, cancer cell markers; COL1A1, ACTA2 (encoding α-SMA), fibroblast markers, CD1C (encoding BDCA1), CD1A, dendritic cell markers; CD14, CD68, macrophage markers; CD3D, CD8A, T cell markers, VWF, EMCN (encoding Endomucin), endothelial cell markers. Clusters were assigned an identity to a given cluster based on expression of these markers. DE genes for each cluster were identified using the “FindAllMarkers” command in Seurat. The cancer cell fraction was subset from the other cell types for further analysis using “Subset” function and then was re-clustered by above functions and DE genes were identified by “FindAllMarkers” command. The sample group separated UMAPs version of the clustering and gene transcripts were performed by “split.by” command and colocalization of two gene transcripts were performed by a “blend = True” command.

### Ingenuity Pathway Analysis (IPA)

DE genes of each cluster were calculated by the “FindAllMarkers” command and written out as “.csv” files in Seurat. To identify the potential upstream regulators and ingenuity canonical pathways, DE genes with a p_val <10^5^ and avg_logFC >1 were loaded into IPA client (QIAGEN) and the analysis were based on the gene ID, expression FC (avg_logFC) and p_val. Ingenuity Canonical Pathways and Upstream Regulators were exported from the IPA analysis and top 15 pathways and upstream regulators were visualized by bar plots.

### Statistical Analysis

The cell number in each cluster of the PDAC primary and metastatic cancer cells were counted by “table” function in Seurat. The cell percentages were calculated and were plotted using GraphPad Prism 8. Wilcoxon Signed-rank test was used for the bioinformatics statistical analysis between different groups and p_values were calculated accordingly.

## Results

### Identification of Major Cell Components in Both Primary and Metastatic PDAC

**Figure 1:**
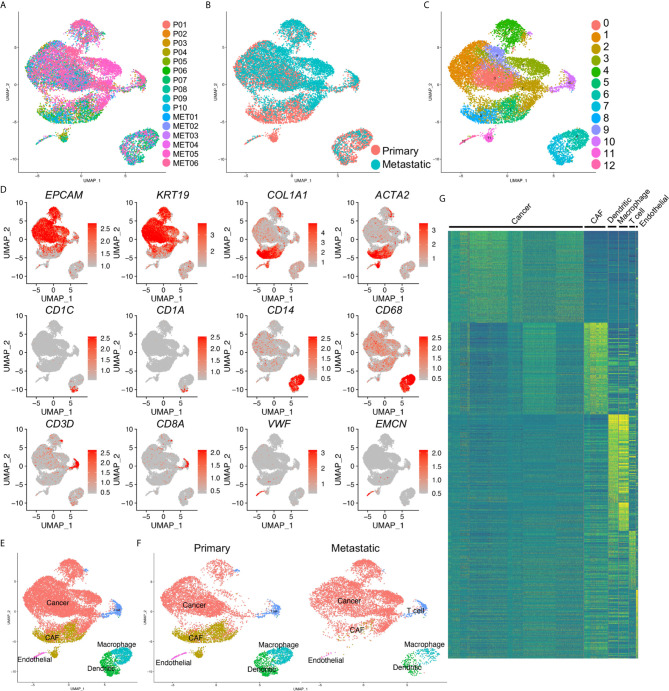
Identification of cancer cells in both primary and metastatic PDAC. **(A)** scRNA-seq data of ten primary (P0–P10) and metastatic (MET0–MET6) PDAC tumors were integrated. **(B)** Primary and metastatic PDAC samples were visualized by UMAP grouped by disease status. **(C)** The cells in the integrated data were divided into thirteen clusters (Clusters 0–12). **(D)** Transcripts of major cell type marker genes were visualized by UMAPs. **(E)** Six distinct cell types were defined based on the expression of cell type marker in **(D)**. **(F)** Cell type of primary and metastatic PDAC by divided UMAPs. **(G)** Heatmap of top 100 genes of each cell type confirmed the distinct signatures of each defined cell type. CAF, cancer associated fibroblasts; cancer, cancer cell, dendritic, dendritic cells.

Multiple cell types were involved in the development and metastasis of PDAC. To identify the cell components in primary and metastatic PDAC, we accessed a recently published data set, which profiled ten primary PDAC tumor tissues and six metastatic PDAC tumor tissues by single cell RNA-sequencing (scRNA-seq) (19). We first integrated all the scRNA-seq samples and corrected the batch effects. The cell qualities in each sample were visualized by nFeature_RNA, nCount_RNA and percent_MT (**Supplemental Figure 1A**, see method literature) and the doublets and high percent_MT apoptosis cells were gated out (**Supplemental Figure 1B**). The distribution of each sample was presented by UMAP. Although some cells showed biased distribution in a few clusters of the integrated data, suggesting different cells numbers in some samples in these clusters, most of the cells were evenly distributed (**Figure 1A**). Also, we checked the samples distribution by their disease status, primary or metastatic, and also found unbiased distribution of most of the cells (**Figure 1B**). Then we clustered the cells and identified thirteen major cell clusters in the integrated data (**Figure 1C**).

To well define the cell types of the clusters, we checked the transcripts of the major cells type markers. *EPCAM* and *KRT19* were common epithelial and cancer cell markers; collagen genes, including *COL1A1* and *ACTA2* (encoding α-SMA protein) were cancer associated fibroblasts (CAF) and myofibroblasts markers; *CD1C* (encoding BDCA1 protein), *CD1A*, were dendritic cell markers; *CD14*, *CD68* were common macrophage markers; *CD3D*, *CD8A* were used as T cell markers, *VWF*, *EMCN* (encoding ENDOMUCIN) were known endothelial cell markers. These marker genes showed specific expression in the clusters of the integrated data (**Figure 1D**), and we accordingly defined them as cancer cells, CAFs, dendritic cells, macrophages, T cells and endothelial cells (**Figure 1E**). Notably, as the previously reported, CAFs could not be detected in the metastatic PDAC tumors, we identified small clusters of dendritic cells and endothelial cells in the metastatic PDAC tumors (**Figure 1F**). The marker gene transcripts visualized by the split UMAP version confirmed the identification of these clusters (**Supplemental Figure 2**). Surprisingly, we could not identify a distinct Epithelial-Mesenchymal-Transition (EMT) cluster in either primary or metastatic PDAC tumors, as we could not found a cluster with both cancer cell and CAF signature gene expression (**Figures 1D–F**, **Supplemental Figure 2**). Heatmap of top 100 genes of each cluster cells confirmed the distinct gene expression patterns of the clusters identified above (**Figure 1G**).

### Differential Gene Expression Programs of the Primary and Metastatic PDAC Cancer Cells

**Figure 2:**
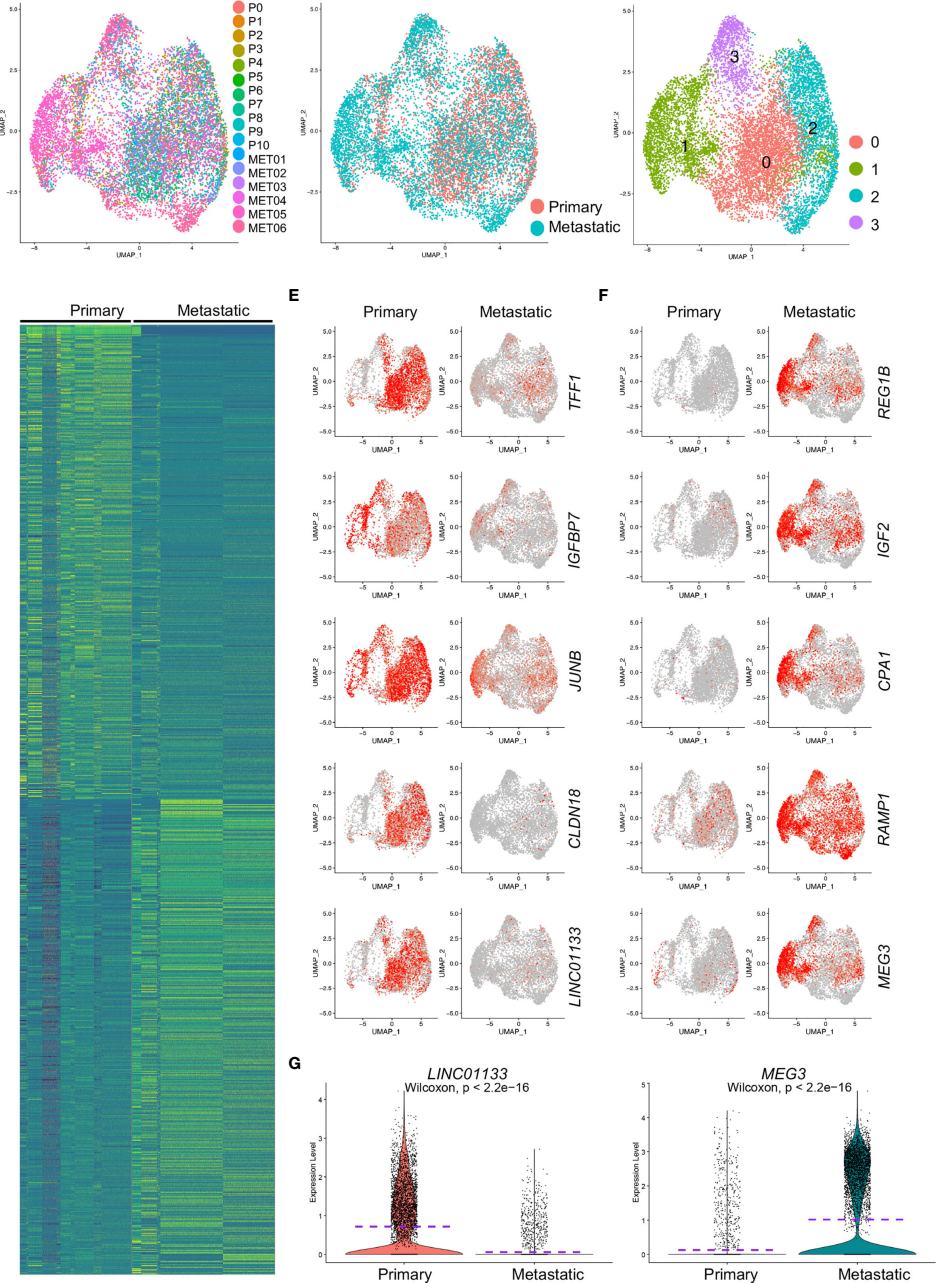
Differentially expressed gene analysis of cancer cells from primary and metastatic PDAC. Cancer cells were extracted and clustered. **(A)** The distributions of each tumor sample were visualized. **(B)** The distribution of primary and metastatic PDAC cancer cells in extracted cancer cell data. **(C)** Cancer cell were clustered, and four clusters (Clusters 0–3) were identified. Comparative analysis was performed to calculate the DE genes between primary and metastatic PDAC cells. **(D)** Top 500 DE genes of both primary and metastatic PDAC cancer cells were visualized by heatmap. **(E, F)** Expression of representative top five genes of primary **(E)** and metastatic **(F)** PDAC cancer cells was visualized by UMAPs to compare their expression in primary and metastatic PDAC cancer cells. Notably, lincRNAs, *LINC01133* and *MEG3*, showed specific expression in primary and metastatic PDAC cancer cells, respectively. **(G)** Violin plot visualization of expression levels of *LINC01133* and *MEG3* expression in primary and metastatic PDAC cancer cells. Dash purple lines indicated the Mean levels of gene expression.

To identify the differentially expressed gene between primary and metastatic PDAC cancer cells, we extracted and re-clustered the cancer cells from the integrated data. Similarly, most of the cells from different samples (**Figure 2A**) or from different disease status (**Figure 2B**) were unbiased distributed. The cancer cells were divided into four major subclusters: Clusters 0–3 (**Figure 2C**). We then performed comparative analysis for the differentially expressed genes between primary and metastatic PDAC cancer cells and input the top genes of both primary and metastatic PDAC cancer cells for heatmap. Heatmap of top 500 genes displayed specific differentiation gene expression patterns (**Figure 2D**).

We visualized five of the top genes of both primary and metastatic cancer cells respectively by UMAPs. *TFF1*, *IGFBP7*, *JUNB*, *CLDN18* and *LINC01133* genes were among the top genes of the primary cancer cells (**Figure 2E**). *TFF1* is a member of the trefoil family and was reported to stimulated both pancreatic cancer and stellate cells and increases metastasis (21). Low expression of gene *IGFBP7* was believed to be associated with poor outcome of pancreatic ductal adenocarcinoma (22). *JUNB* gene encodes a transcriptional factor, and it regulated the expression of *FAM83A*, a gene significantly elevated in human and murine pancreatic cancers (23). *CLDN18* (Claudin-18), a member of Claudin family, is a gastric epithelium–associated claudin and was reported as an early-stage marker of pancreatic carcinogenesis (24). Five representative top genes significantly upregulated in metastatic cancer cells were also visualized by violin plots (**Figure 2F**). Regenerating proteins, including REG1A and REG1B, promoted acinar-to-ductal metaplasia and acted as novel diagnostic and prognostic markers in pancreatic ductal adenocarcinoma (25). Ontological interaction network analysis highlighted the dysregulation of a set of carboxypeptidase family proteins including carboxypeptidase A1 (CPA1) in PDAC (26) and a genome sequencing study identified an association of *CPA1* gene with Familial Pancreatic Cancer (FPC) (27). Although most of the genes we identified here, except the two LincRNAs, *LINC01133* and *MEG3*, were closely associated with and some even were believed to be biomarkers of PDAC, a comprehensive regulation network and core regulators were not yet determined. Taken the facts that LincRNAs are a special class of genes that control gene expression programs on multiple levels thereby contributing to cancer progression and that LINC01133 and *MEG3* were attractively differentially expressed in primary and metastatic PDAC cancer cells (**Figure 2G**), we revealed the hypothesis that these two LincRNAs were crucial regulators of PDAC cancer cell metastasis.

### *MEG3* and LINC01133 Showed Predominant Expression in Distinct Cancer Cell Clusters

**Figure 3:**
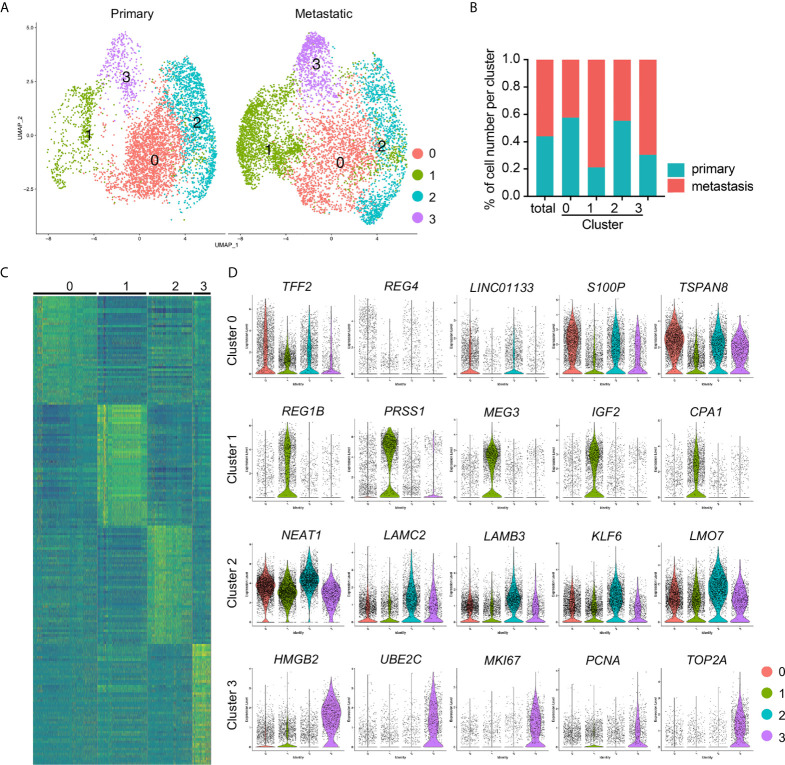
Identification of cancer cell subclusters PDAC cancer cells. **(A)** Cell clusters of primary and metastatic PDAC cancer cells were visualized by spilt UMAPs. **(B)** Percentage of the primary and metastatic cancer cells in each cluster. **(C)** Heatmap of top 50 genes of each cluster. **(D)** Presenting of relative expression of representative top genes of each cancer cell cluster. Notably, lincRNAs, *LINC01133*, *MEG3* and *NEAT1*, were the top genes of Clusters 0, 1 and 2, respectively. Cluster 3 exhibited significantly increased expression of proliferating marker genes.

Cancer cell in the integrated data were divided into four distinct subclusters and some clusters showed unbiased cell contribution from primary and metastatic PDAC (**Figure 3A**), although batch effects was removed. We then counted the cell numbers of each cancer status in each cluster and found comparable total cancer cell number from primary and metastatic PDAC. However, most of cancer cells in Cluster 1 (78.90%) were from metastatic PDAC, and Cluster 0 and Cluster 2 included almost comparable cell number from primary and metastatic PDAC (**Figure 3B**).

To determine the gene profiles of each cancer cluster, we performed differentially expressed gene analysis and identified significant differentially expressed genes. We input the top 50 genes of each cluster to generate a heatmap, which confirmed the distinct gene expression pattern of each cluster (**Figure 3C**). We presented five of the representative top genes by violin plots and found significantly differential expression of these genes in different clusters (**Figure 3D**). More interestingly, most of the signature genes in Cluster 1 were same to those of metastatic cancer cells (**Figures 2F**, **3D**), including *REG1B*, *MEG3* and *CPA1*, this was possibly because of the major cell contribution of this cluster from metastatic PDAC. However, although Cluster 0 contained comparable cell number from primary and metastatic cancer, some of its signatures were similar to those of primary PDAC cancer cells (**Figures 2E**, **3D**), *LINC01133* and trefoil family member gene *TFF2*, for example. Although the signatures of Cluster 2 were not as specific as the other three clusters, as most of the top genes displayed some background in other clusters, significantly increased expressed genes could easily be identified in this cluster. The most significantly differentially expressed gene was LincRNA, *NEAT1*, and other genes included Laminin gene family members, *LAMC2* and *LAMB3*, Kruppel Like Factor family member, *KLF6*, and *LMO7* (**Figure 3D**). Cluster 3 was highlighted by the significantly increased transcripts of proliferating marker genes, including *MKI67*, *PCNA* and DNA Topoisomerase gene, *TOP2A*, suggesting activated cell proliferation in this cancer cell cluster.

**Figure 4:**
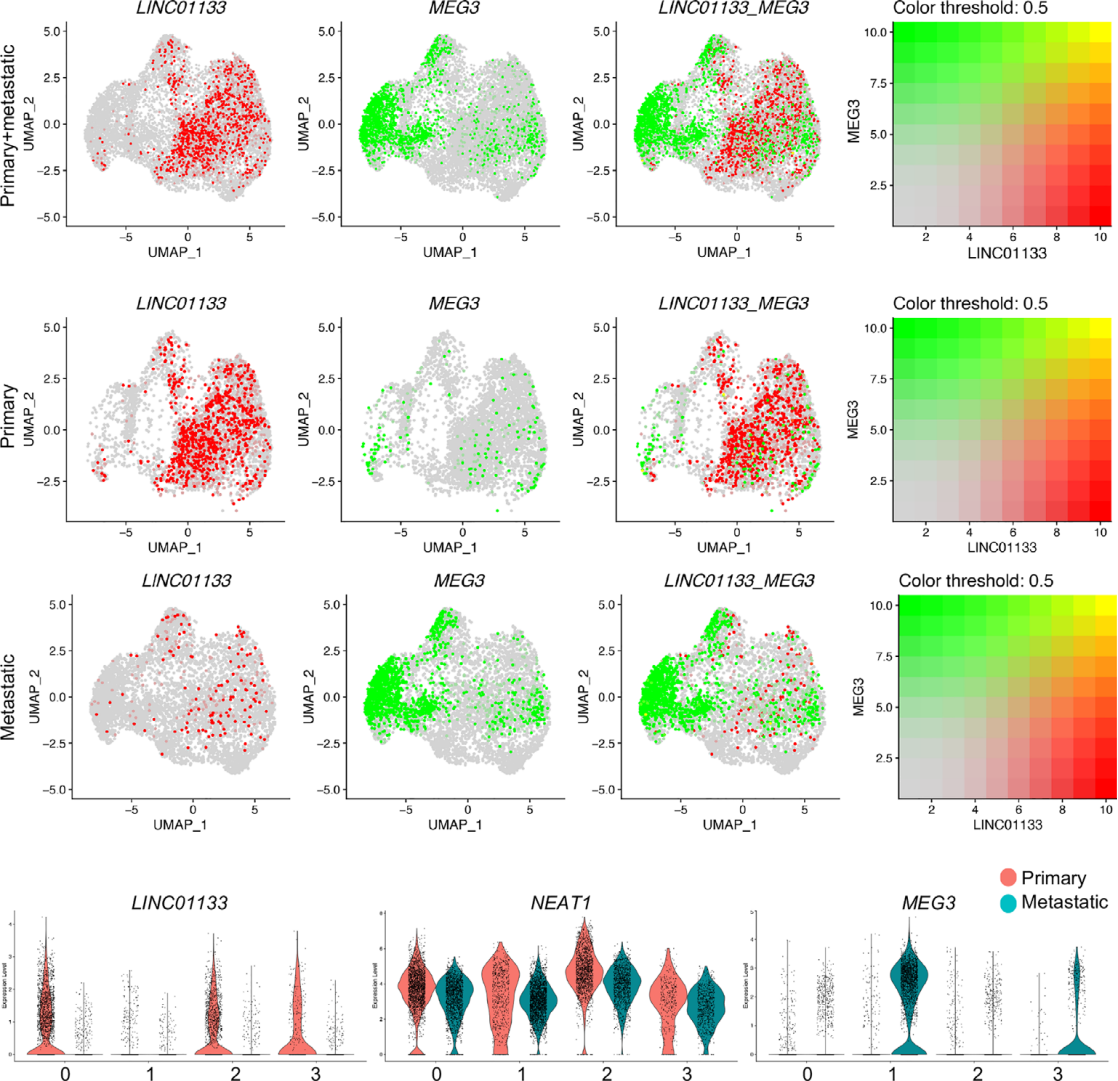
*LINC01133* and *MEG3* marked primary and metastatic PDAC cancer cell clusters, respectively. **(A)** Relative expression and colocalization of *LINC01133* and *MEG3* in integrated cancer cells, primary and metastatic cancer cells by blended UMAPs. **(B)** Comparison of *LINC01133* and *MEG3* expression in each cluster between primary and metastatic cancer cells by split violin plots. Notably, *MEG3^+^* cancer cells were mostly distributed in Cluster 1 and derived from metastatic PDAC cancer, while *LINC01133 ^+^* cancer cells were mostly distributed in Clusters 2 and 3 and derived from primary PDAC cancer.

Among these cluster specific genes, the most interesting ones, again, were the LincRNAs, *LINC01133*, *MEG3* and *NEAT1*, each individual of which represented one individual cluster respectively. To better check the specification of these genes in each cluster of each sample, we co-localized their transcripts by the blend UMAPs in the integrated cancer cell data and split primary and metastatic cancer cells. *LINC01133* positive and *MEG3* positive cells were well separated in the integrated cancer cell data and showed little overlap (**Figures 3D**, **4A**). In split cancer cells, little *MEG3* transcript in primary cancer cells and little *LINC01133* transcript in metastatic cancer cells were detected. Most of the *LINC01133* transcript positive cells were in primary Custers 0 and 2, while most of the MEG3 positive cells were in metastatic Cluster 1, and little overlap were found (**Figure 4A**). Although *NEAT1* showed most condensed transcript in Cluster 2, it was also detectable in Clusters 1 and 3, and overlapped a little bit with *MEG3* expression in metastatic Cluster 1 (**Supplemental Figure 3A**) and exhibited a good overlap with LINC01133 transcript in primary Cluster 3 (**Supplemental Figure 3B**). Violin plots split by the cancer status further confirmed the above observations (**Figure 4B**).

During the preparation of this manuscript, a latest report selected *MEG3* as the potential target gene due to decreased *MEG3* expression in PDAC tissues compared to normal tissues in several microarray datasets (28). To explain the inconsistency, we checked the expression of *MEG3* and the other two LincRNAs, *NEAT1* and *LINC01133*, in the scRNA-seq data of all cell components of primary and metastatic PDAC tumors. We found significantly increased expression of *MEG3* in the CAFs but little expression in cancer cells of the primary tumors, as well as remarkable transcript in cancer cells in metastatic PDAC tumors (**Supplemental Figure 4**). This possibly could explain the inconsistency, as the microarray data of previous study were performed on the tumor tissues composed of multiple cell components. Otherwise, *MEG3* was playing diverse roles in PDAC development and metastasis, which needed to be confirmed by further investigation. The other lincRNAs, *LINC01133* displayed specific expression in primary cancer cells, while *NEAT1* was abundant in most of the cell components of both primary and metastatic PDAC tumors (**Supplemental Figures 4B–D**).

### Differentiation Potentials of the Cancer Cell Clusters

**Figure 5:**
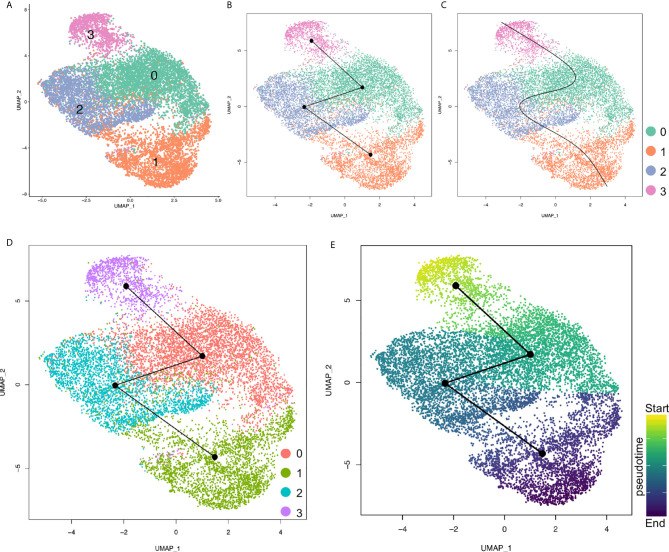
Pseudotime analysis of cancer cell clusters with Slingshot. **(A)** Cancer cells were clustered under Slingshot packages and similar clusters were identified. **(B, C)** Inferred trajectory **(B)** and principal curves of each lineage **(C)** were visualized by UMAP. **(D)** Cluster information from Seurat were mapped to Slingshot and both analysis assays were matched. **(E)** Pseudotime values for each cancer cell cluster were visualized by UMAP. Color depth of each cell/cluster were correlated with pseudotime values.

To determine the differentiation potentials of the cancer cells from primary and metastatic PDAC tumors, we loaded the cancer cells into Slingshot packages, which provides unified interface to dozens of different trajectory inference methods *via* docker containers (29), for pseudotime analysis. The cancer cells were also segregated into four major distinct clusters (**Figure 5A**). Analysis on inferred trajectory and principal curves demonstrated smoothed representations of each subcluster (**Figures 5B, C** **)**. To validate the cluster definition, we mapped the clusters identified by Seurat and found matched cluster information of the two analysis methods (**Figures 5A–D**). Further pseudotime analysis confirmed high differentiation potentials of Cluster 3 (proliferating cluster) and that Cluster 1 was at the end of the principal curves, while Clusters 0 and 2 were in the middle. This analysis suggested that less metastatic clusters (Clusters 0 and 3) were mostly derived from proliferating cluster (Cluster 3), and finally differentiated into metastatic cluster (Cluster 1) (**Figures 5E**).

### Elevated *MEG3* in Cancer Cells of PDAC Mouse Models

**Figure 6:**
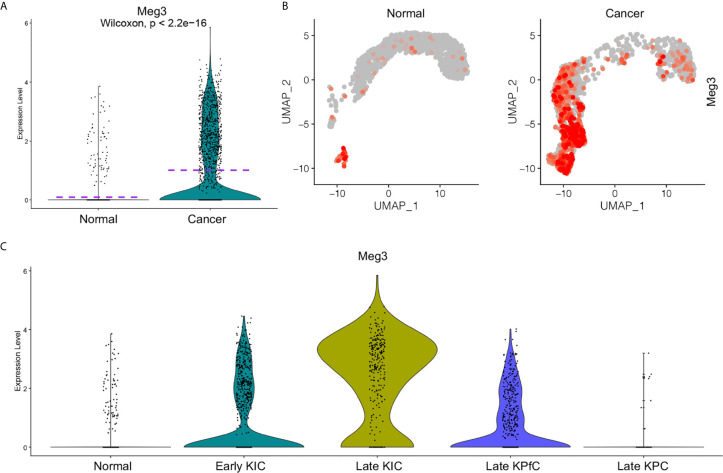
Elevated *MEG3* transcript in cancer cells of PDAC mouse models (33). Transcript levels of *MEG3* in normal epithelial cells and PDAC cancer cells of mouse models were visualized by split violin plots **(A)** and UMAPs **(B)**. **(C)** Upregulated *MEG3* expression in cancer cells of PDAC mouse models including early KIC, late KIC and late KPfC compared to normal epithelial cells. Rare *MEG3* was detected in late KPC due to extremely low cell number.

To further study the functions of *MEG3* in the progression of PDAC, we re-accessed the scRNA-seq data (17) on three commonly used PDAC mouse models (KIC, *Kras^LSL−G12D/+^Ink4a^fl/fl^Ptf1a^Cre/+^*; KPfC, *Kras^LSL−G12D/+^Trp53^fl/fl^Pdx1^Cre/+^*; KPC, *Kras^LSL−G12D/+^Trp53^LSL−R172H/+^Ptf1a^Cre/+^*) (30, 31) and normal pancreatic tissues from a recently published study. We integrated the scRNA-seq data on different samples (**Supplemental Figure 5A**) and clustered the cells of the integrated data (**Supplemental Figure 5B**). By checking the epithelial cell lineage marker gene expression in the integrated data (**Supplemental Figure 5C**) (32), the epithelial/cancer cell clusters were identified (**Supplemental Figure 5D**) and extracted for re-clustering (**Supplemental Figure 5E**). *Meg3* showed rare transcript in normal epithelial cell but was significantly elevated in the cancer cells of PDAC mouse models with the exception of Late KPC due to extremely low cell number in this model (**Supplemental Figure 5F**). Comparative analysis confirmed that significant upregulation of *Meg3* transcription in cancer cells of PDAC mouse models compared to normal epithelial cells (**Figures 6A, B**). Significantly, *Meg3* transcript levels increased at early stage of KIC mouse model and was even remarkably elevated in late stage of KIC mouse model (**Figure 6C**), suggesting a close involvement of *Meg3* in PDAC development.

### Increased EMT Signatures in *MEG3* Positive Metastatic Cancer Cells

**Figure 7:**
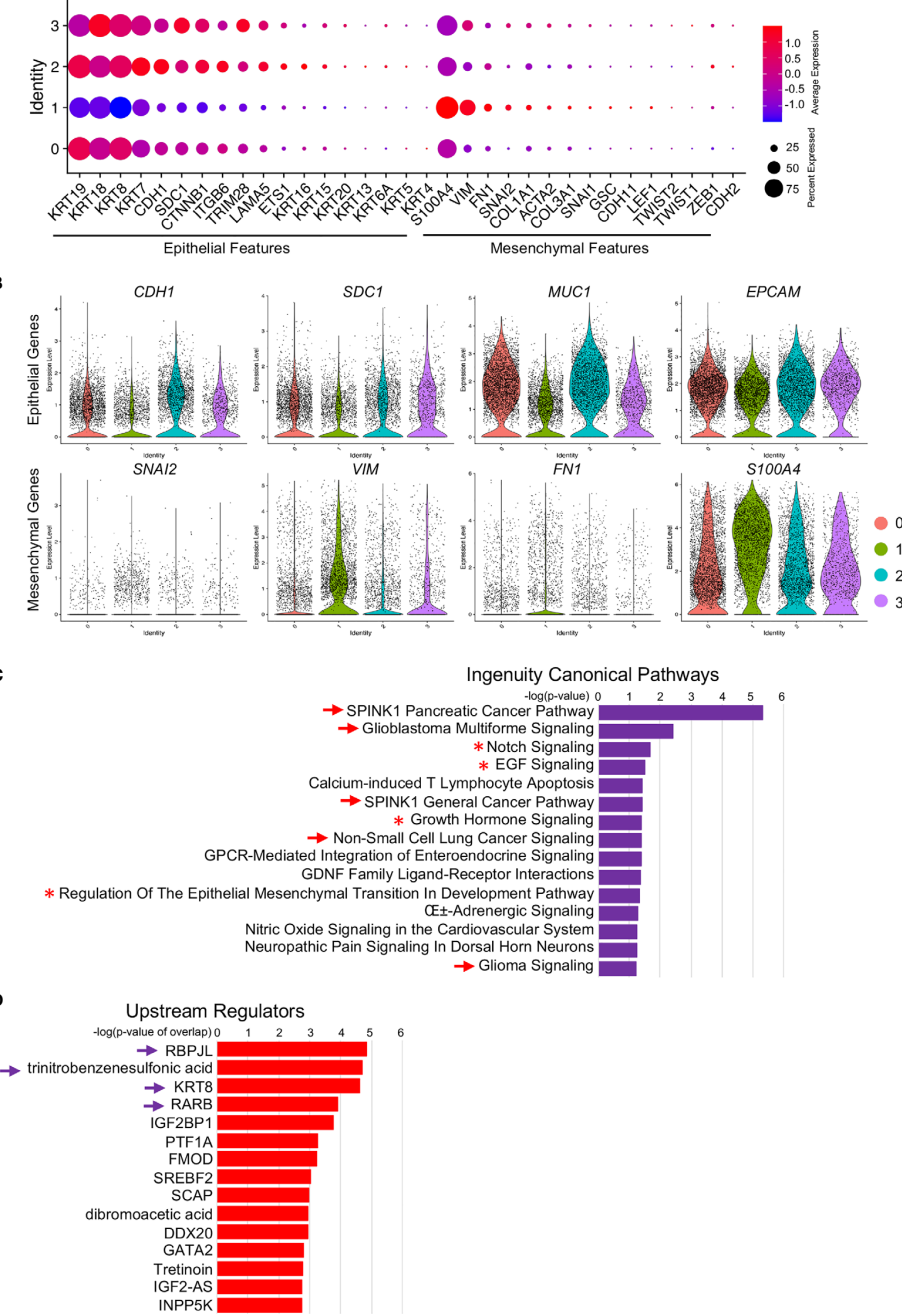
Increased EMT signatures in *MEG3* ^+^ cancer cells. **(A)** Relative expression of EMT markers, including epithelial markers and mesenchymal markers, in each cluster were visualized by dot plot. **(B)** Decreased epithelial cell marker signatures and increased mesenchymal marker signatures in Cluster 1 were confirmed by violin plot visualization of representative EMT marker expression: *CDH1*, *SDC1*, *MUC1*, *EPCAM*, *SNAI2*, *VIM*, *FN1* and *S100A4*. **(C, D)** Ingenuity Canonical Pathways **(C)** and Upstream Regulators **(D)** of Cluster 1 determined by IPA on significant DE genes. The arrow and arrowhead indicated signaling pathways and upstream regulators were cancer or cancer metastasis related. Asterisk indicated the EMT signaling pathway.

By these scRNA-seq transcript data, we identified four signatured cancer cell clusters, one of which was mostly derived from metastatic PDAC tumors and marked by *MEG3* transcripts. To investigate the potential functions of this metastatic cluster, we further studied the differentially expressed genes of this cluster and found significantly activated Epithelial–Mesenchymal-Transition (EMT) related signatures, indicated by increased mesenchymal related gene expression and decreased epithelial related gene expression (**Figure 7A**). We visualized four of the representative genes by violin plots and found reduced expression of *CDH1* (E-Cadherin encoding gene), *SDC1* (Syndecan-1 encoding gene), *MUC1* (Mucin1 encoding gene) and *EPCAM*, and upregulated expression of *SNAI2* (Slug encoding gene), *VIM* (Vimentin encoding gene), *FN1* (Fibronectin1 encoding gene) and *S100A4* (FSP1 encoding gene) (**Figure 7B**). These gene transcripts were further validated by UMAPs (**Supplemental Figure 6A**) and split UMAPs confirmed the downregulated epithelial marker genes expression and upregulated mesenchymal marker expression in the Cluster 1 (**Supplemental Figures 6B, C** **)**. Many other oncogenes (**Supplemental Figure 6D**) and tumor suppressor genes (**Supplemental Figure 6E** **)** involved in pancreatic cancer progression and metastasis were also visualized by violin plots. For example, SPINK1 in pancreatic cancer has several potentially important clinical applications ranging from a biomarker to a potential new target for cancer therapy (34). SPINK1 showed abundant expression in both primary and metastatic cancer cells but was significantly elevated in metastatic cancer cells (**Supplemental Figure 6D**). Tumor suppressor gene FOLR1 encodes a protein FRα, the high expression of which in surgically removed PDAC specimens was found to be significantly associated with favorable prognosis (35). FOLR1 transcript was remarkably reduced in metastatic cancer cells compared to primary cells (**Supplemental Figure 6E**). Many other genes were also well involved biologically and clinically.

To validate the functions and potential related signaling pathways of this *MEG* ^+^ metastatic cancer cell cluster, we input the significant differentially expressed genes (**Supplemental Table 1**, genes at: avg_logFC >1 and p_value <10^5^) of this cluster into IPA and identified several malignant cancer related signaling pathways (**Figure 7C**, arrows), including SPINK1 Pancreatic Cancer Pathway and Glioblastoma Multiforme Signaling, as well as EMT related signaling pathways, Notch Signaling (36), EGF Signaling (37), Growth Hormone Signaling (38) and Regulation Of The Epithelial Mesenchymal Transition In Development Pathway (**Figure 6C**, asterisks, **Supplemental Table 2**). We also identified several upstream regulators and many of them were closely related to the oncogenesis and progression of PDAC (39), chronic pancreatic disease (40) and PDAC related epithelial to mesenchymal plasticity (41) (**Figure 7D**, arrows, **Supplemental Table 2**). Differentially expressed genes of LINC01133 marked Cluster 0 were also input into IPA and the top Ingenuity Canonical Pathways and Upstream Regulators were also presented. Top pathways, including Oxidative Phosphorylation, EIF2 Signaling and Mitochondrial Dysfunction, were closely associated with stem and immunoevasive properties (33), tumor biology (42) and cancer metabolic phenotypes (43) of pancreatic cancer. Taken together, these ingenuity canonical pathways and upstream regulator analysis consistently confirmed our observation in scRNA-seq data and further identified the *MEG* ^+^ metastatic cancer cell cluster as the leader of cancer cell EMT and metastasis.

## Discussion

PDAC represents a cancer entity of extraordinarily high malignancy, particularly poor prognosis, and constantly increasing patient numbers. Its aggressive biology and the fact that most patients present in advanced or disseminated stages of disease render the development of novel PDAC treatment strategies one of the superordinate challenges in current oncological research (44). In spite of these clinical manifestations, another challenge of PDAC research community is the intra-tumoral cellular heterogeneity. In particular, its heterogeneity is presented not only by the intra-tumoral cell diversity, including stroma, inflammatory cells constituting high percentage of the tumor mass embedded with normal pancreatic tissue due to the infiltrative nature of PDAC (45), but also by the heterogeneity of the cancer cells themselves in primary and metastatic PDAC tumors. This extensive degree of heterogeneity makes it rather challenging to identify genetic variants based on bulk mRNA sequencing (46). Here in this current study, by accessing a recent scRNA-seq dataset on primary and metastatic PDAC tumors, we profiled the primary and metastatic PDAC cancer cells and revealed their differentially expressed gene programs. In addition to the validation of commonly studied protein gene targets, we also identified two specific LincRNAs, *MEG3* and LINC01133, which specifically represented one of the cancer cell clusters respectively. LINC001133 was predominantly expressed in a less aggressive cluster in primary cancer cells, while *MEG3* was primarily expressed in a metastatic cancer cell cluster, which displayed increased EMT signatures and was potentially leading cancer cell metastasis.

With the development and use of single cell transcriptomics technologies, transcriptomic profiling at a single-cell resolution enables quantitative measurements of the molecular activity that underlies the phenotypic diversity of cells within a tumor (47). Advancements in high-throughput sequencing and imaging technologies provide opportunities to identify and characterize diverse cancer types with intra-tumor heterogeneity (48), like pancreatic cancer. A recent study developed and applied a multiplexing strategy in which cells from 198 cancer cell lines of 22 pan-cancer types were profiled in pools by scRNA-seq and then computationally assigned to the corresponding cell lines. Twelve expression programs were identified that were recurrently heterogeneous within multiple cancer cell lines and the programs were associated with diverse biological processes, including cell cycle, senescence, stress and interferon responses, EMT and protein metabolism. Most of these programs recapitulated those recently identified as heterogeneous within human tumors (49).

Even more studies are targeted on the clinically dissect cancer tumors of pan-cancers and corresponding non-tumor tissues adjacent to cancer (18, 46), which revealed the genetics and epigenetic programs related to vivo cancer cell and environment activities. By employing scRNA-seq to acquire the transcriptomic atlas of individual pancreatic cells from primary PDAC tumors and control pancreases, diverse malignant and stromal cell types, including two ductal subtypes with abnormal and malignant gene expression profiles were respectively identified in PDAC. The heterogenous malignant subtype was found to be composed of several subpopulations with differential proliferative and migratory potentials. Cell trajectory analysis revealed that components of multiple tumor-related pathways and transcription factors were differentially expressed along PDAC progression. A subset of ductal cells with unique proliferative features were associated with an inactivation state in tumor-infiltrating T cells, providing novel markers for the prediction of antitumor immune response and suggesting potential biomarkers for anticancer treatment such as targeted therapy and immunotherapy (46). Another study performed time course scRNA-seq experiments of human PDAC, as well as mouse PDAC model induced by the expression of Kras-G12D (50). Similarly, the scRNA-seq data also revealed the heterogeneity of acinar metaplastic cell types and their potential interactions with immune and stromal cells. These findings shed light on the sequence of events that led to acinar cell transformation and revealed several metaplastic cell types and states, from which malignant cells could develop. In addition, this study described the transcriptional changes of fibroblast, endothelial cells and immune cells during the development of the disease and revealed some new potential markers for early detection of PDAC (18). Further studies were achieved by scRNA-seq analysis of patient-derived PDAC organoids by a recent pre-print study. Single-cell transcriptome analysis of PDAC organoids and primary PDAC identified distinct tumor cell states shared across patients, including a cycling progenitor cell state and a differentiated secretory state. These cell states are connected by a differentiation hierarchy, with ‘classical’ subtype cells concentrated at the endpoint of this hierarchy. In an imaging-based drug screen, expression of ‘classical’ subtype genes also correlates with better response to clinical drugs. These scRNA-seq based results uncovered a functional hierarchy of PDAC cell states linked to transcriptional tumor subtypes and supported the use of PDAC organoids as a clinically relevant model for *in vitro* studies of tumor heterogeneity (51).

These single cell transcriptomic profiling studies at a single-cell resolution in cancer offers the opportunity to identify and characterize transcriptionally distinct subpopulations and states that may impact clinical outcomes, inform treatment strategies, or point to new therapeutic opportunities (15, 47). However, all these studies focused on the comparative analysis on primary PDAC and non-tumor tissue. As another important feature of PDAC is its early progression to metastatic disease (1), a transcriptomic study gap between the disease statuses existed. A most single-cell transcriptome analysis of PDAC primary tumors and metastatic lesions revealed distinct cell types in primary and metastatic PDAC tissues including tumor cells, CAFs, endothelial cells and immune cells (19). The expression levels of cell type-specific markers for cancer cells, activated CAFs, and endothelial cells were significantly associated with patient survival (19). To further deepen this study and reveal the metastasis regulation programs of PDAC, in the current study, we accessed the scRNA-seq dataset and determined the DE gene programs of the PDAC primary tumors and metastatic lesions. We defined a heterogeneous feature of the PDAC cancer cells and identified the major subclusters of the cancer cells. Notably, each subtype was represented by a specific LincRNA expression and the most aggressive subcluster was mostly derived from metastatic PDAC. This subcluster, specifically marked by the expression of *MEG3*, showed significantly increased signatures of EMT and displayed a leading potential in cancer cell metastasis.

This LincRNA gene, *MEG3*, was closely involved in modulating drug resistance to chemotherapy in multiple types of human cancers including pancreatic cancer (52). Bulk levels of *MEG3* expression were negatively correlated with PI3K expression and were closely correlated with tumor size, metastasis and vascular invasion in pancreatic cancer (53). Total expression of *MEG3* was not statistically correlated to either histological grade or tumor node metastasis stage in the 25 cases of micro-dissected pancreatic cancer tissues (54), which we proposed was due to the unpurified cell they used. *MEG3* is involved in modulating drug resistance to chemotherapy in multiple types of human cancers (52). During the preparation of this manuscript, a latest study selected *MEG3* as the potential target gene by combination and reuse of several microarray datasets on PDAC and normal tissues and found decreased *MEG3* in PDAC tissues (28). Some other studies used other experimental methods on bulk tissues including qPCR (53) and gene edition on cancer cell lines (54, 55) revealed a suppressing effect of *MEG3* on human pancreatic cancer. These data were promising and informative, but some concerns arose due to the complex cell components in the PDAC tissues of these bulk array data and most of the *in vitro* validation data depended too much on cancer cell lines, which were known to have many limitations and not be able to fully mimic the transcriptional natures of human cancers (56, 57). The advancements of the current study were that we targeted *MEG3* at single cell levels of purified cancer cells, and these cells were at the natural stages of *in vivo* tumors. By transcriptional profiling of these cells, a distinct metastatic cancer cell cluster was identified with specific *MEG3* transcription, suggestion a different *in vivo* function of *MEG3* in PDAC cancer cell metastasis with previous studies. Or else, *MEG3* was conducting a diverse role in the development and metastasis of PDAC, respectively, when coordinating our data on primary and metastatic PDAC tissues at single cell levels with the above discussed studies. But further investigation on this inconsistence is highly necessary and full of interest. In conclusion, these LincRNA genes and their marked clusters are like to be novel biological and clinical targets to study the development and metastasis of PDAC and is going to attract a broad audience in pan-cancer community.

## Data Availability Statement

The original contributions presented in the study are included in the article/**Supplementary Material**. Further inquiries can be directed to the corresponding author.

## Author Contributions

CW conceived the study. HP and HD analyzed the data. HP and CW prepared the manuscript, and WZ, TW, and PW reviewed the result and the discussion of the manuscript. All authors contributed to the article and approved the submitted version.

## Funding

This work was supported by grants from the National Natural Science Foundation of Liaoning Province (No. 20180530081 to CW) and Young and middle-aged scientific and technological talents support program of Shenyang City (No. RC200554 to CW).

## Conflict of Interest

The authors declare that the research was conducted in the absence of any commercial or financial relationships that could be construed as a potential conflict of interest.
