# Supplementary material for: A Cancer Cell Cluster Marked by LincRNA MEG3 Leads Pancreatic Ductal Adenocarcinoma Metastasis
Source: Front Oncol. 2021 May 13;11:656564. doi: 10.3389/fonc.2021.656564 (PMC8155708; doi:10.3389/fonc.2021.656564)
Supplement: Supplementary file 1 [file DataSheet_1.pdf]

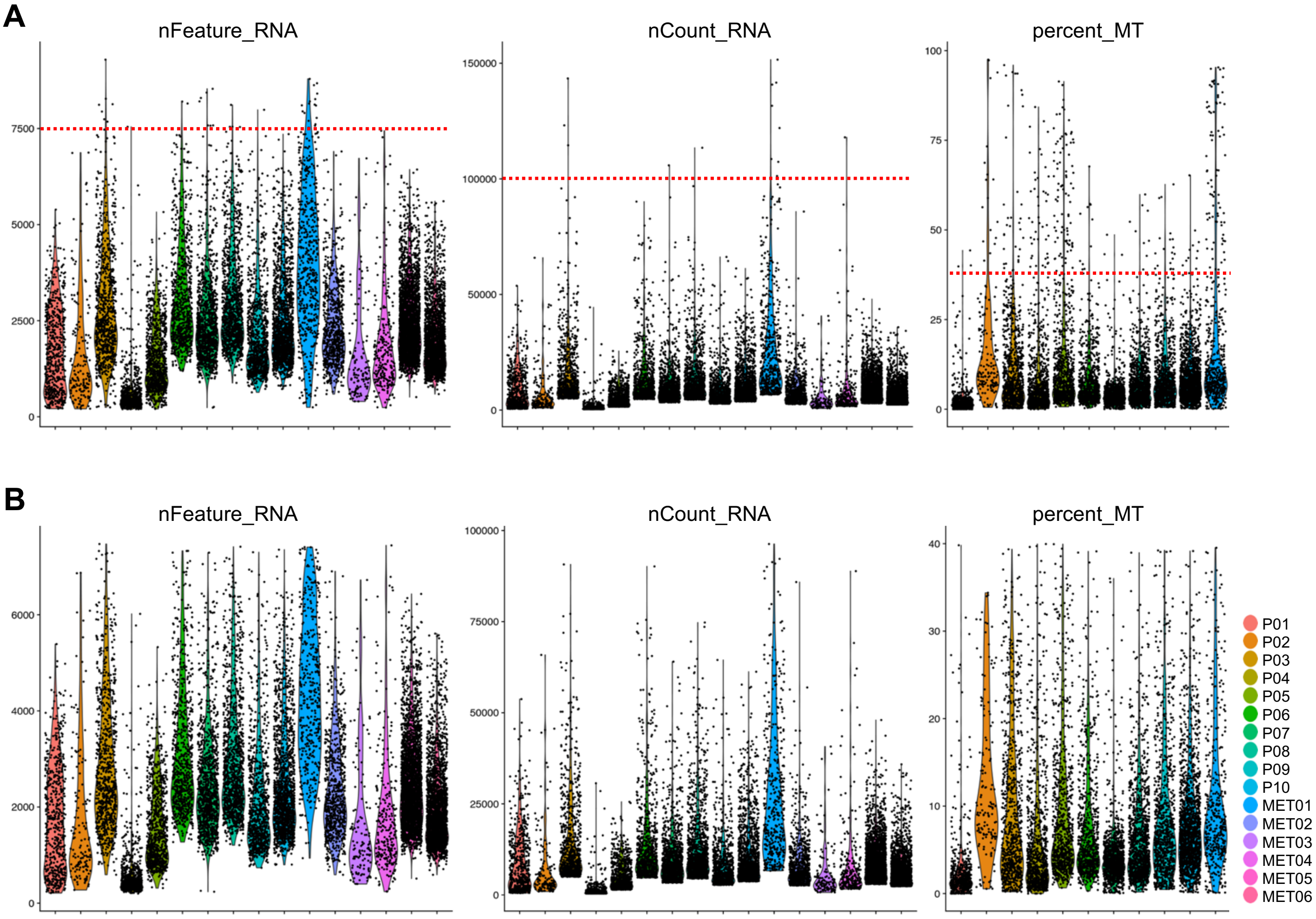

Supplemental Figure 1 | Quality control (QC) of single-cell RNA-seq data. Violin plots of the total number of genes detected each cell (nFeature_RNA), number of transcripts each cell (nCount_RNA), and percentage of mitochondrial genes each cell (percent.MT) of each single cell RNA-seq sample before **(A)** and after **(B)** QC. Quality control was performed by removing low-quality cells and doublets by subsetting the cells at nFeature_RNA < 7500 & nCount_RNA < 100000 & percent.mt < 40: Red dish lines.

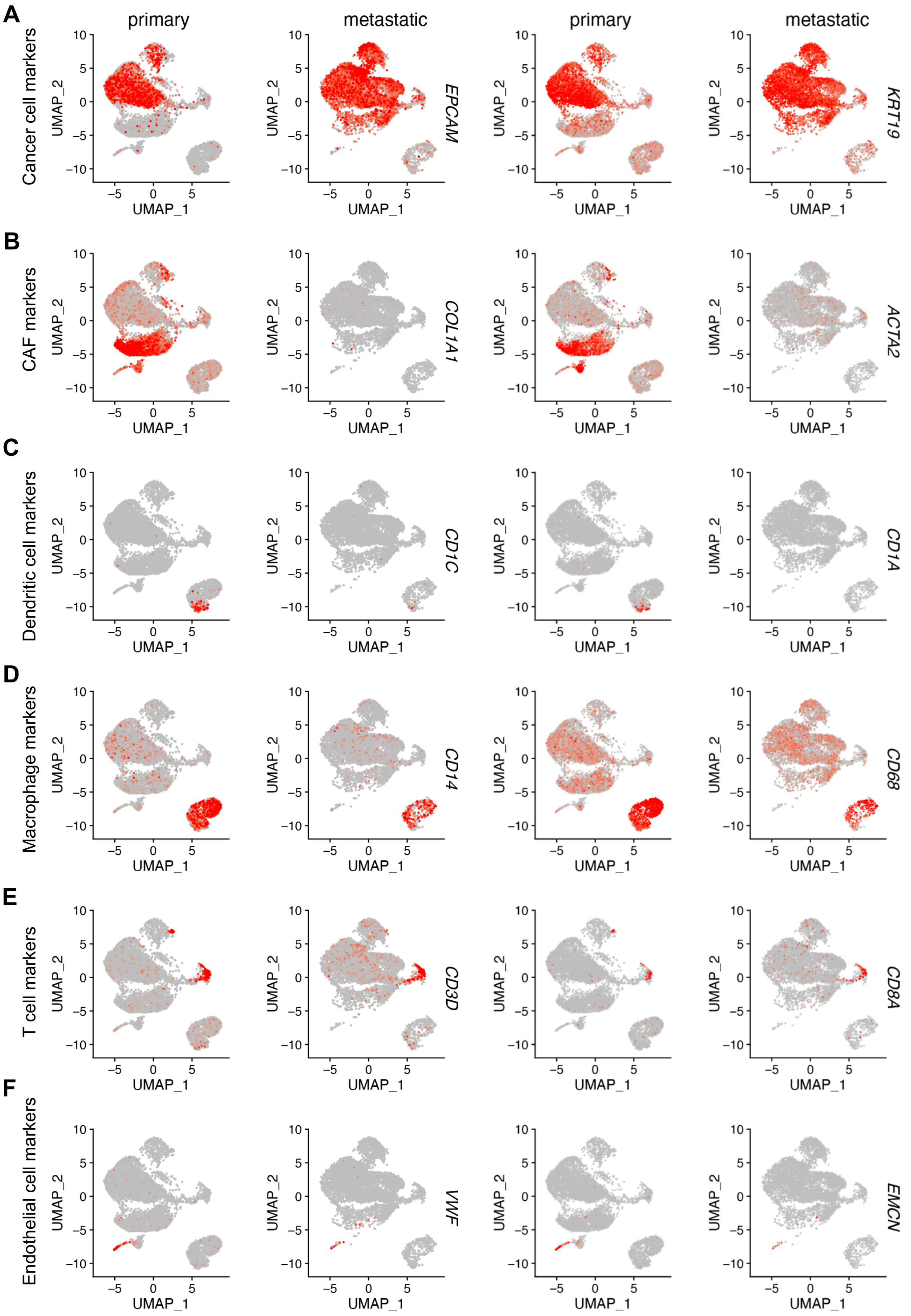

Supplemental Figure 2 | Cell type marker expression in scRNA-seq data of primary and metastatic PDAC. Canonical cell type marker gene expression was visualized by UMAP: *EPCAM*, *KRT19*, cancer cell markers **(A)**; *COL1A1*, *ACTA2*, fibroblast markers **(B)**; *CD1C*, *CD1A*, dendritic cell markers **(C)**; *CD14*, *CD68*, macrophage markers **(D)**; *CD3D*, *CD8A*, T cell markers **(E)**; *VWF*, *EMCN*, endothelial cell markers **(F)**. Notably, dendritic cell and endothelial cell clusters were also identified in metastatic PDAC cancer cells, although cell numbers were very low.

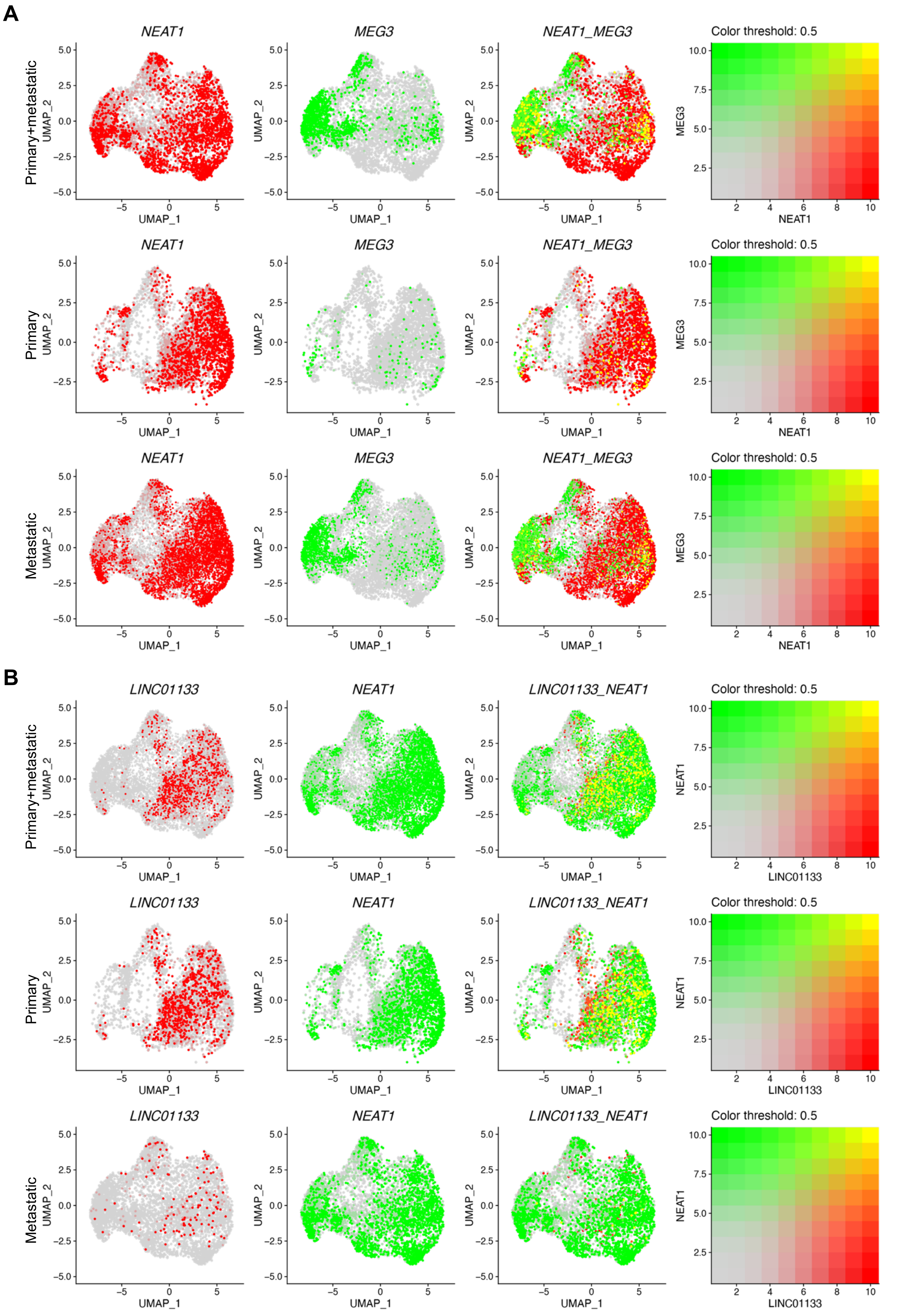

Supplemental Figure 3 | Colocalization of lincRNAs, *NEAT1*, *MEG3* and *LINC01133* in clusters of primary and metastatic PDAC cancer cells. **(A)** Distribution of *NEAT1* and *MEG3* positive cells in integrated, primary and metastatic PDAC cancer cells. MEG3 was mostly expressed in Cluster 1 in metastatic cancer cells. NEAT1 showed wide expression in primary and metastatic PDAC clusters but was predominantly expressed in Cluster 2 and Cluster 3. **(B)** Colocalization of *LINC01155* and *NEAT1* in integrated cancer cells, primary and metastatic PDAC cancer cells. *LINC01155* and *NEAT1* showed some overlap but *LINC01155* showed more specification in Cluster 2 and Cluster 3 of primary PDAC cancer cells.

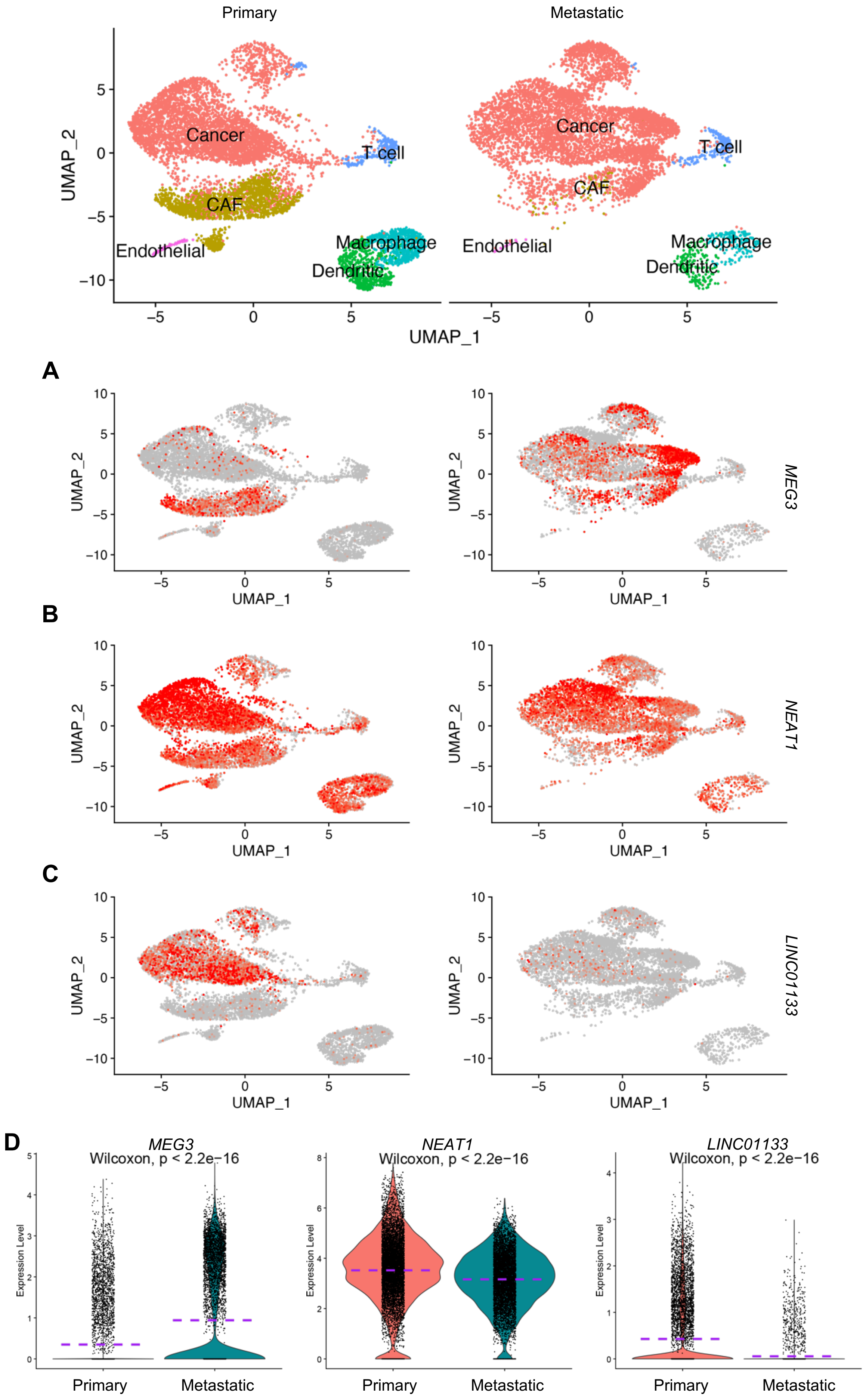

Supplemental Figure 4 | Transcripts of related LincRNAs in other cell components of primary and metastatic PDAC tumors. The transcript levels of *MEG3* **(A)**, NEAT1 **(B)** and *LINC01133* **(C)** in the scRNA-seq of total cells of primary and metastatic PDAC tumors were visualized by split UMAPs. Notably, the expression of *MEG3* was extremely high in CAFs in primary PDAC tumors, while *LINC01133* was specifically expressed in cancer cells of metastatic PDAC tumors. **(D)** Transcript levels of *MEG3*, NEAT1 and *LINC01133* in total cells of primary and metastatic PDAC tumors. Dash purple lines indicated the Mean levels of gene expression.

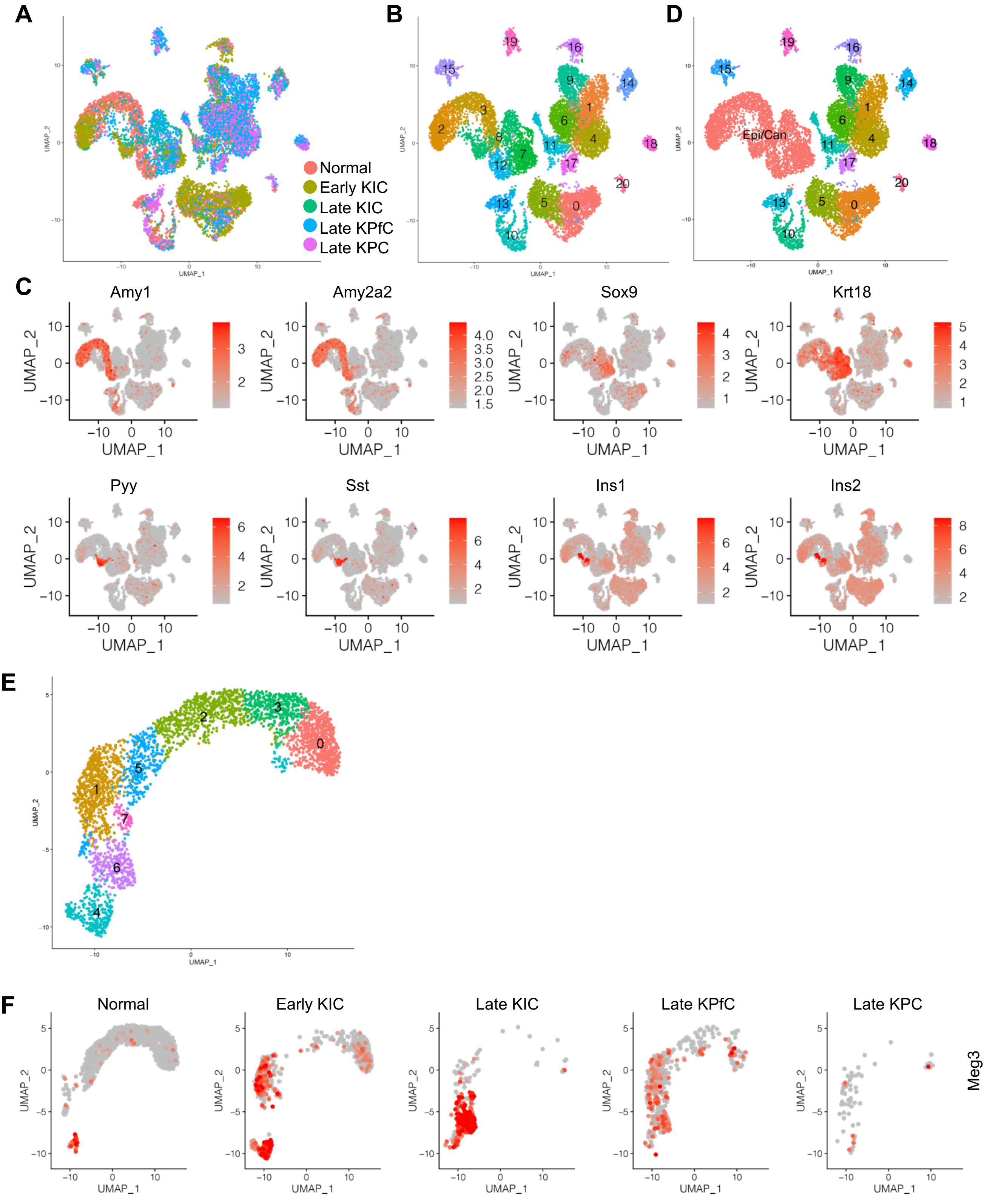

Supplemental Figure 5 | **s**cRNA-seq analysis of cancer cells from PDAC mouse models. **(A-B)** Integration of scRNA-seq data on normal and cancer tissues (**A**) and clusters identification in the integrated data (**B**). (**C-D**) Epithelial/Cancer cells cluster identification (**D**) based on the epithelial lineage markers gene expression (**C**). (**E**) The epithelial/cancer cells were extracted and clustered. (**F**) Transcript of Meg3 in the epithelial/cancer cells of each mouse model.

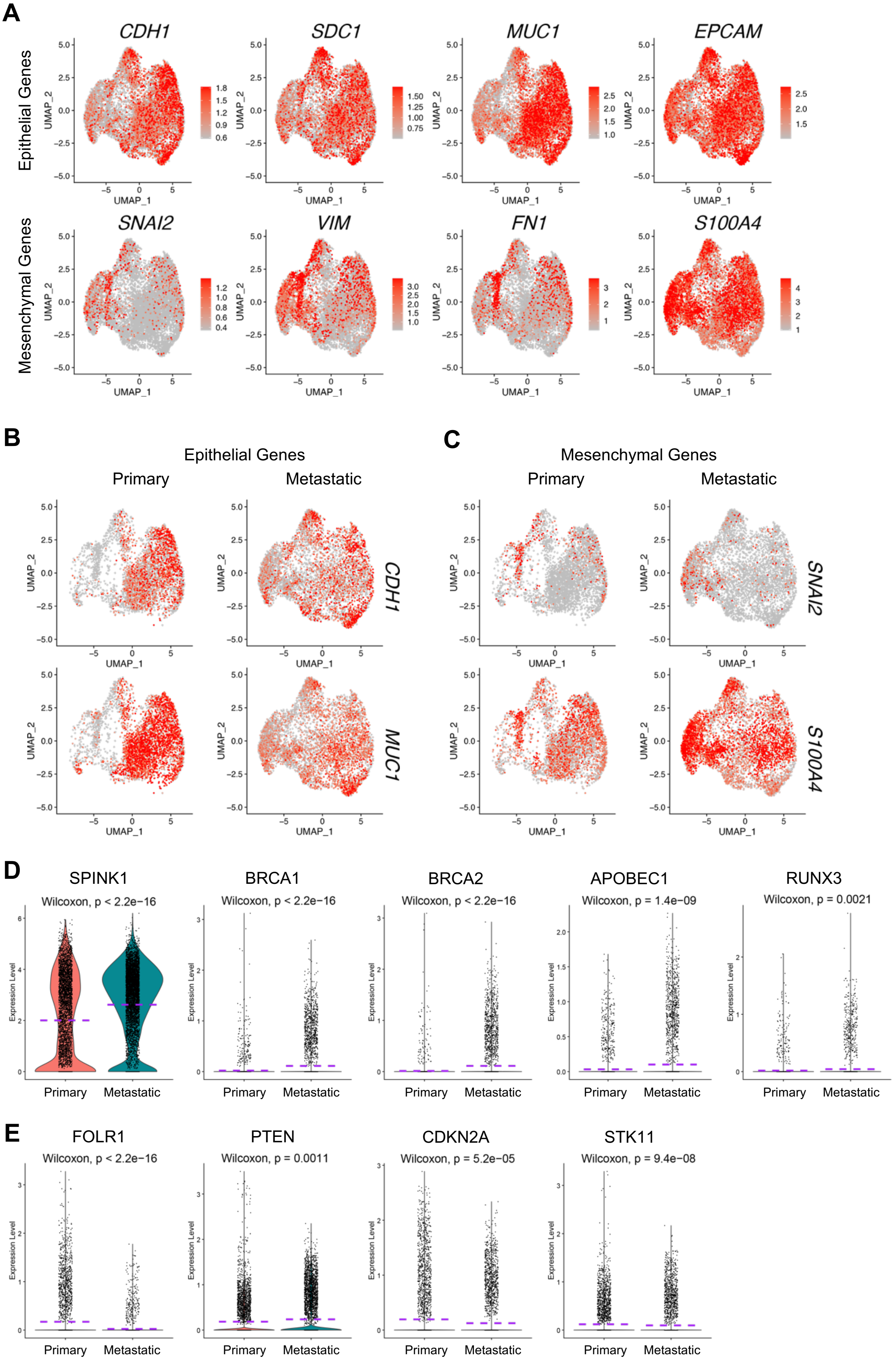

Supplemental Figure 6 | Transcripts of EMT markers in primary and metastatic PDAC cancer cells. **(A)** Relative expression of representative EMT markers in integrated PDAC cancer cells. **(B, C)** Relative expression of representative epithelial **(B)** and mesenchymal **(C)** markers in integrated primary and metastatic PDAC cancer cells. Metastatic PDAC cancer cells showed increased overall mesenchymal markers and transcripts of the mesenchymal markers were mainly contributed by Cluster 1. (**D-E**) Violin plot comparison of commonly reported oncogenes (**D**) and tumor suppressor genes (**E**) of pancreatic cancer. Dash purple lines indicated the Mean levels of gene expression.

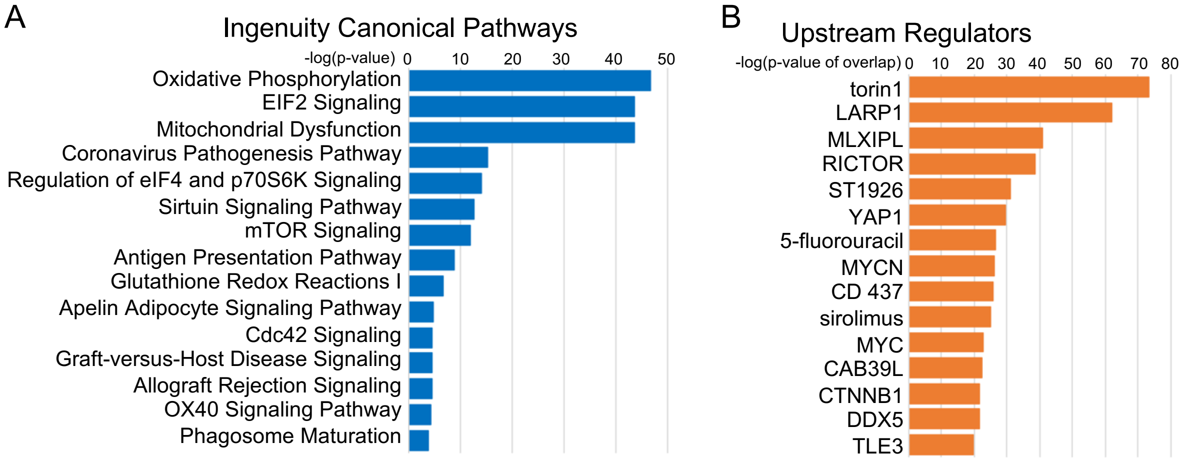

Supplemental Figure 7 | IPA analysis of specific genes of LINC01133 marked cluster 0. Ingenuity Canonical Pathways **(A)** and Upstream Regulators **(B)** of Cluster 0 determined by IPA on significant DE genes.
